# Identification of genomic differences among peripheral arterial beds in atherosclerotic and healthy arteries

**DOI:** 10.1038/s41598-018-22292-y

**Published:** 2018-03-02

**Authors:** Marja Steenman, Olivier Espitia, Blandine Maurel, Beatrice Guyomarch, Marie-Françoise Heymann, Marc-Antoine Pistorius, Benjamin Ory, Dominique Heymann, Rémi Houlgatte, Yann Gouëffic, Thibaut Quillard

**Affiliations:** 1 UMR1238 INSERM, Université de Nantes, CHU de Nantes, Nantes, France; 2 l’institut du thorax, INSERM, CNRS, UNIV Nantes, Nantes, France; 3 Department of Internal Medicine, CHU de Nantes, Nantes, France; 4 Department of Vascular Surgery, CHU de Nantes, Nantes, France; 5 INSERM U1256, NGERE, University of Nancy, Nancy, France; 6 DRCI, University Hospital of Nancy, Nancy, France; 7 Department of Oncology and Metabolism, University of Sheffield, INSERM, European Associated Laboratory “Sarcoma Research Unit”, Sheffield, UK; 8 Institut de Cancérologie de l’Ouest, INSERM, U1232, Université de Nantes, Nantes, France

## Abstract

Calcification is independently associated with cardiovascular events and morbidity. The calcification burden in atherosclerotic lesions quantitatively and qualitatively differs between arterial beds. Cardiovascular risk factors (CVRF) differentially affect plaque development between arterial beds. The aim of this study was to evaluate the impact of CVRF on atherosclerotic plaque calcification and to further study the molecular arterial heterogeneity that could account for these differences. Histological analysis was performed on atherosclerotic plaques from 153 carotid, 97 femoral and 28 infrapopliteal arteries. CVRF showed minor associations with plaque calcification: age and hypertension affected only the overall presence of calcification but not the type of the calcification, which significantly differed between arterial beds. Transcriptome analysis revealed distinct gene expression profiles associated with each territory in atherosclerotic and healthy arteries. Canonical pathway analysis showed the preferential involvement of immune system-related processes in both atherosclerotic and healthy carotid arteries. Bone development-related genes were among those mostly enriched in atherosclerotic and healthy femoral arteries, which are more prone to developing endochondral calcification. This study highlights the heterogeneous nature of arteries from different peripheral vascular beds and contributes to a better understanding of atherosclerosis formation and evolution.

## Introduction

Vascular calcification is an independent predicting factor for cardiovascular events and morbidity^1^. Vascular calcification is associated with a worse prognosis after lower limb artery endovascular revascularization: multivariate analysis reported that the percentage of calcified plaque is an independent predictor of binary restenosis at 12 months^2^. Vascular calcification favors plaque rupture and contributes to hypertension depending on its localization and extent.

Anatomo-histological studies have shown that atherosclerotic plaque compositions largely differ between anatomical locations in peripheral arterial diseases (PAD). Plaque calcifications are heterogeneous with various types of calcifications, including predominantly microcalcifications in carotid arteries (CA) and bone tissue (osteoid metaplasia) in femoral arteries (FA)^3,4^. These differences do not derive from distinct stages of plaque progression, as femoral plaques tend to develop later than those in CA^5^. The discrepancies in calcification burden could therefore derive from different shear stress conditions^6^, intrinsic biological differences between vascular cells as suggested by their diverse embryological origins^7,8^, or exposure to different cardiovascular risk factors (CVRF), as CVRF also differentially affect plaque development differentially between arterial beds^9–12^.

The CVRF for PAD mirror those of cerebrovascular and coronary atherosclerosis, including a positive family history, diabetes mellitus, smoking, chronic kidney disease, hypertension, dyslipidemia and age^12–18^. Smoking and diabetes are particularly virulent and are independently associated with worse outcomes^19^. The influence of CVRF on atherosclerotic localization is well known^9–11^ with a strong relationship between smoking or dyslipidemia, whereas diabetes appears more specific to below the knee disease^11^ and hypertension favors intracranial atherosclerosis^9^.

The differential impact of systemic CVRF on vascular beds and differential atherosclerotic plaque calcifications and compositions suggest biological arterial heterogeneity. Numerous clinical data support this concept^20–23^. Molecular data supporting biological heterogeneity in human peripheral arteries are scarce, however. Seo *et al*. compared gene expression in proximal and distal aorta from heart donors^24^. They identified a small group of genes with location-dependent expression levels. More recently, Sulkava *et al*. identified genes differentially expressed between atherosclerotic plaques from CA and FA and from abdominal aortas in humans, reflecting plaque heterogeneity between these beds^25^.

The aim of our study was to better understand differential plaque calcification among different peripheral arterial beds. We first analyzed the association between CVRF exposition and atherosclerosis plaque calcification using our human plaque bio-collections ECLA and ECLAGEN. As classic CRVF did not show a major impact on plaque calcifications in CA, FA or infrapopliteal arteries (IPA), we analyzed arterial-bed-specific gene expression profiles in both atherosclerotic and healthy arteries by transcriptomic analysis with a particular focus on calcification-associated genes.

## Materials and Methods

### Patients

From February 2008 to December 2015, atheromatous plaques were harvested and collected (ECLA and ECLAGEN bio-collections) from patients undergoing carotid, femoral or infrapopliteal endarterectomy in the Department of Vascular Surgery at Nantes University Hospital. Details about this bio-collection have been published elsewhere^26,27^. Healthy arteries free of atherosclerotic lesions were obtained from organ donors. Sample collection and handling was performed in accordance with the guidelines of the Medical and Ethical Committee in Nantes, France, and written informed consent was obtained from all patients and from next of kin for all organ donors. The experimental protocol was approved by the Agence de Biomédecine (research protocol #PFS09–014, authorized on Dec 23, 2009, by the Agence de Biomédecine, France). Legal and ethical authorizations were granted by the French Research Ministry (n° DC-2008–402), the National Commission for Computerized Information and Liberties (CNIL, n° 1520735 v 0), and the local ethical committee (GNEDS). Patients suffering from non-atherosclerotic peripheral arterial disease, thrombosis or restenosis were excluded. Demographic and clinical data were collected, including age, gender, treatment, CVRF (high blood pressure, diabetes mellitus, dyslipidemia, tobacco use (active/past user) and obesity (BMI ≥ 30 kg/m^2^)), and Cockcroft creatinine clearance. Prior to surgery, blood specimens were collected for lipid balance and phospho-calcic metabolism assessments.

CVRF were defined as follows. Regarding smoking, we unfortunately do not know whether smoking was still active at the sampling time. We only looked at tobacco exposure. The definition of diabetes was the presence of two fasting blood glucose measurements >1.26 g/l. Hypertension was defined as the presence of persistent blood pressure (>140 mmHg diastolic, 90 mmHg systolic) for more than three months. Dyslipidemia was present when LDL levels were higher than defined thresholds, which depended on the presence of a cardiovascular event, diabetes, and the number of associated CVRF.

For CA and FA, endarterectomies were performed on a consecutive series of patients using conventional surgical techniques according ESVS guidelines^28^. For these arteries, the sample was limited to one lesion because endarterectomies were performed. The plaque was removed at the bifurcation from the lumen as a single specimen. For IPA, the sample with the most severe atheromatous lesion was harvested. All samples were 1–2 cm long. For histology, we analyzed sections of the core of the lesion present in each arterial sample.

CA samples were mostly collected in endarterectomy procedures (97.4% of cases), and arterial bypass was performed in 2.6% of cases. For FA, 68.4% of the arteries were removed during bypass surgery, 30.5% after endarterectomy and 1.1% after amputation. Approximately 11.6% of the FA were thrombosed before the surgical procedure. For IPA, 88% were removed after amputation and 12% after bypass surgery. Approximately 24% of IPA were thrombosed before the surgical procedure.

The exclusion criteria for non-atheromatous arteries and patients were a history of cardiovascular diseases (ischemia cardiopathy, stroke, or peripheral artery diseases) or the presence of macroscopic athero-thrombosis during tissue collection.

### Histology processing

The atherosclerotic plaques were harvested and fixed in 10% formalin for 24–48 h and then decalcified in Sakura TDE 30 fluid. They were embedded in paraffin. Sections (4-µm thickness) were stained with hematoxylin eosin (HE). Whole sections were imaged with a NanoZoomer digital slide scanner (Hamamatsu Photonics, Hamamatsu, Japan).

### Histological classification of atherosclerotic plaque calcification

The sections were graded according to a previously described strategy^3,4^. Atherosclerotic plaque calcification classification was based on five categories: no calcification, microcalcifications, sheet calcifications, nodular calcifications, and osteoid metaplasia^3^. Microcalcifications consisted of <50-µm vesicle-like structures outlined by calcium deposits; sheet calcifications were defined as a large calcifying fibrosis; nodular calcification was characterized by dense circular calcified structures; osteoid metaplasia consisted of bone tissue with typical stratified osteoid matrix encapsulating osteocyte-like cells surrounding lipid-rich bone marrow.

For each artery, three sections of pathological or healthy segments were analyzed. Sections were classified in a blinded fashion by two independent investigators (TQ and OE).

### Gene expression analysis

Samples for RNA processing were harvested and immediately snap-frozen in liquid nitrogen or stored in All-protect Tissue Reagent (Qiagen). Total RNA was extracted from 31 healthy arteries (10 CA, 11 FA, and 10 IPA) and 65 atherosclerotic arteries (27 CA, 25 FA, 13 IPA) using Macherey Nagel NucleoSpin columns (Macherey Nagel, Düren, Germany). RNA was hybridized to Agilent Human Gene Expression Microarrays. Fluorescence values corresponding to raw expression data were extracted using Feature Extraction Software (Agilent). Positive and negative control probes were removed. Non-linear effects, such as background or saturation, were corrected by Lowess against a median profile of all samples. Values of replicate probes were averaged, and the data matrix was filtered to 20,000 probes based on highest median expression values. Microarray data have been deposited in NCBI’s Gene Expression Omnibus (GEO) and are accessible through GEO Series accession number GSE100927. Clusters of co-expressed genes were identified using the partitional clustering method k-means (k = 10)^29^ on natural-log-transformed and gene-median-centered data with uncentered correlation as a similarity metric in Gene Cluster 3.0^30^. Hierarchical clustering was performed using Gene Cluster 3.0, and heatmaps were displayed using Java Treeview^31^. Clusters separating the different arterial beds were selected, and a collective p was calculated. For each sample, a mean expression value of all genes from the cluster was calculated. The hereby obtained mean values of different arterial beds were compared. This strategy, which was based on strong correlations of gene expression, allowed us to avoid multitesting.

Two-class significance analysis of microarrays (SAM)^32^ was used to identify genes with statistically significant differential expression between different arterial territories. With this method, each gene is first assigned a score on the basis of a modified t-test, and genes with scores greater than a user-defined threshold are selected. Repeated random sample permutations are used to estimate the percentage of genes identified by chance (false discovery rate, FDR) among the selected genes. The FDR is similar to a p adjusted for multiple comparisons. For all SAM analyses, the chosen delta threshold corresponded to the lowest median FDR (FDR = 0%).

Gene Ontology (GO)^33^ enrichment analysis of the different clusters was performed using GoMiner^34^. Enrichment of GO terms was determined using the 20,000 probe list as background. Annotations with FDR < 0.05 were considered significant. GO terms enriched in gene lists obtained after SAM analysis were filtered to remove redundancy, and the top scoring elements were visualized using the web-based tool REVIGO (http://revigo.irb.hr/)^35^. For atherosclerotic arteries, only GO terms with FDR = 0% were used as input in REVIGO. For healthy arteries, GO terms with FDR < 0.05 were used. The parameters were as follows: Allowed similarity = Small (0.5); Homo sapiens database; and Simrel as semantic similarity measure.

### Statistical analysis

Continuous data are presented as the mean ( ± standard deviation). Categorical variables are presented as counts (proportions). T-tests were performed to test for significant differences in continuous parameters between two or more groups. The χ2 or Fisher exact test (based on expected frequency) was used to compare categorical variables between groups. The Bonferroni method was used for post hoc tests. We adjusted the p level according to the number of hypotheses tested. Logistic regression analysis [with odds ratios (OR) and confidence intervals (CI)] was used to evaluate the association between CVRF and calcification. From univariate analysis, we selected variables with p < 0.10 (statistical criterion). Variables were eliminated from highest to lowest p in the multivariate model, but remained in the final model if p was less than 0.05 or seemed to be confounding (more than 10% change in estimate). All two-way interactions between pairs of predictors in the model were tested one at a time. p < 0.05 was considered statistically significant when no Bonferroni correction was applied. Data were analyzed with SAS packages (SAS Institute Inc. version 9.4, Cary, NC).

CVRF and calcifications were analyzed by comparing the arterial territories (CA, FA and IPA) in pathological arteries.

### Data availability statement

The datasets generated and/or analyzed in the current study are available from the corresponding author upon reasonable request.

## Results

### Cardiovascular risk factors, arterial territories and atherosclerotic plaque calcification heterogeneity

Two hundred seventy-eight atherosclerotic arteries were evaluated (153 CA, 97 FA and 28 IPA). We first assessed the impact of CVRF on atherosclerotic disease in different arterial territories (Table 1). Tobacco use was significantly associated with atherosclerotic FA; diabetes mellitus was more frequent in patients with diseased IPA. Dyslipidemia was more frequent in carotid disease, and patients with infrapopliteal disease were significantly older and had a lower estimated glomerular filtration rate (eGFR) under 60 ml/min than patients with disease in other territories.

**Table 1:** Frequency of cardiovascular risk factors among arterials beds. (CA: carotid arteries, FA: femoral arteries, IPA: infrapopliteal arteries, eGFR: estimated glomerular filtration rate).

	Tobacco n, (%)	Hypertension n, (%)	Diabetes mellitus n, (%)	Dyslipidemia n, (%)	Mean age ( ± SD)	Sex (male) n, (%)	Obesity n, (%)	eGFR < 60 ml/min n, (%)
**CA**	59 (38.6)	123 (80.4)	35 (22.9)	120 (78.9)	70 ± 10	112 (73.2)	22 (14.4)	43 (28.1)
**FA**	50 (51.5)	74 (76.3)	33 (34.0)	66 (68.0)	67 ± 9	85 (87.6)	16 (16.5)	17 (17.5)
**IPA**	2 (7.1)	23 (82.1)	18 (64.3)	12 (42.9)	74 ± 12	21 (75.0)	7 (25.0)	13 (46.4)
**Global p**	<0.001	0.73	<0.001	0.001	0.003	0.02	0.34	0.008
**FA vs CA**	0.05	—	0.06	0.07	0.001	0.007	—	0.07
**FA vs IPA**	<0.001	—	0.008	0.03	0.001	0.13	—	0.005
**CA vs IPA**	0.001	—	<0.001	<0.001	0.06	0.52	—	0.07

Before surgery, patients were treated with statins in 68.6% of CA, 66.3% of FA and 68% of IPA cases. Patients were treated with ACE inhibitor therapy in 61.6% of CA, 31.6% of FA, and 20% of IPA cases. Angiotensin 2 receptor blockers were given to 22.6%, 15.8% and 8% of patients with CA, FA, and IPA, respectively. Patients received another antihypertensive treatment, respectively, in 45.8%, 45.3% and 64% of CA, FA, and IPA cases. Diabetes was treated in 22.9% of patients with CA, 34% of patients with FA and 64% of patients with type 2 diabetes had metformin in 5.9%, 14.7% and 24% of CA, FA, and IPA cases respectively.

The non-atherosclerotic group consisted of 62 patients (20 CA, 20 FA and 22 IPA), the mean age was 49.3 years, 47.3 years and 47.2 years for CA, FA, and IPA, respectively; 72.2% were men (65%, 75% and 77.3%, respectively, in CA, FA, and IPA), 33.9% had current smoking (35%, 35% and 31.8% in CA, FA, and IPA, respectively); 24.2% had hypertension (30%, 20% and 22.7% in CA, FA, and IPA, respectively), 1.6% had diabetes (5% of CA); 9.7% had dyslipidemia (15%, 10% and 4.5% in CA, FA, and IPA, respectively), and 27.4% were obese (35%, 25% and 22.7% in CA, FA, and IPA, respectively). Approximately 6.5% of non-atherosclerotic group had estimated glomerular filtration under 60 ml/min (10% of CA, 5% of FA and none in IPA).

**Figure 1:**
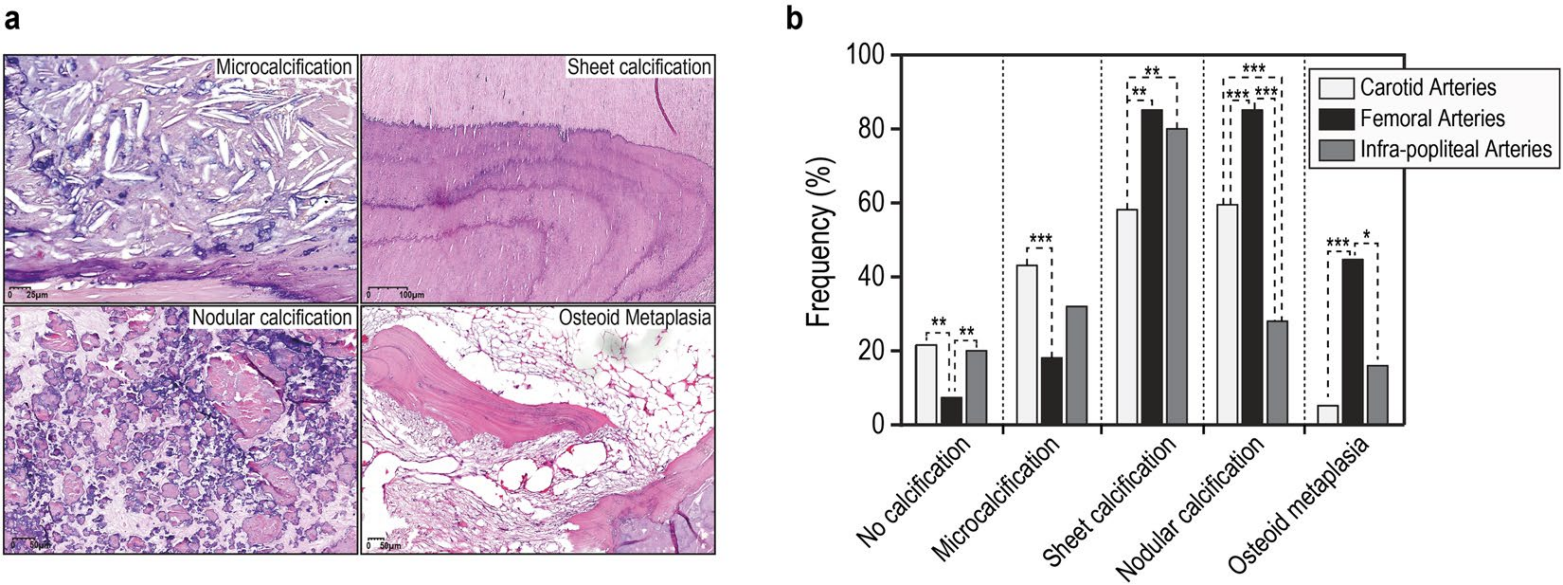
(**a**) Hematoxylin eosin (HE) staining showing microcalcification, sheet calcification, nodular calcification, and osteoid metaplasia. (**b**) Atherosclerotic plaque calcification distribution among arterial territories (*p < 0.05, **p < 0.01, and ***p < 0.001).

All diseased arteries presented with advanced atherosclerotic plaques and differentially encompassed the main calcification types illustrated in Fig. 1a,b shows the distribution of calcification types according to arterial territory.

Overall, FA were extensively calcified and presented more plaque calcification than CA (p = 0.02).

Osteoid metaplasia was more frequent in FA than in CA (p < 0.0001) or IPA (p < 0.05). In contrast, CA developed more microcalcifications than FA (p = 0.001). Sheet and nodular calcifications also varied among arterial beds: FA presented with more sheet (p < 0.0001) and more nodular calcification (p < 0.0001) than CA. Forty-four samples showed no calcification at all. In most cases, several types of calcification were present within the same atherosclerotic plaque. On average, 1.6 types of calcifications were found in CA; 2.6 in FA and 1.5 in IPA.

Multivariate analysis comparing calcified vs non-calcified plaques showed that age (p = 0.003), hypertension (p = 0.02) and territory (p = 0.007) were significantly associated with the presence of calcification in atherosclerotic plaques. However, no significant association was identified between CRVF and type of calcification (osteoid metaplasia, microcalcification, sheet calcification or nodular calcification) (Table 2).

**Table 2:** Frequency of cardiovascular risk factors in calcification subtypes. No significant link was found between risk factors and calcification subtypes. (OM: osteoid metaplasia; SD: standard deviation; eGFR: estimated glomerular filtration rate).

	Microcalcifications n = 91	Sheet n = 189	Nodules n = 178	OM n = 54	No calcification n = 44
Tobacco n, (%)	41 (44.6)	78 (84.8)	74 (80.4)	25 (27.2)	17 (15.6)
Hypertension n, (%)	79 (41.6)	160 (84.2)	145 (76.3)	42 (22.1)	26 (13.2)
Diabetes mellitus n, (%)	28 (39.4)	64 (90.1)	55 (77.5)	20 (28.2)	10 (14.7)
Dyslipidemia n, (%)	67 (40.6)	140 (84.8)	132 (80.0)	40 (24.2)	31 (16.7)
Mean age ( ± SD)	71 ± 10	70 ± 11	72 ± 12	71 ± 10	66 ± 10
Sex (male) n, (%)	69 (36.9)	160 (85.6)	146 (78.1)	43 (23.0)	28 (14.2)
Obesity n, (%)	20 (54.1)	33 (89.2)	29(78.4)	7 (18.9)	6 (15.8)
eGFR < 60 ml/min n, (%)	25 (39.1)	54 (84.4)	44(68.8)	15 (23.4)	7 (11.7)

**Figure 2:**
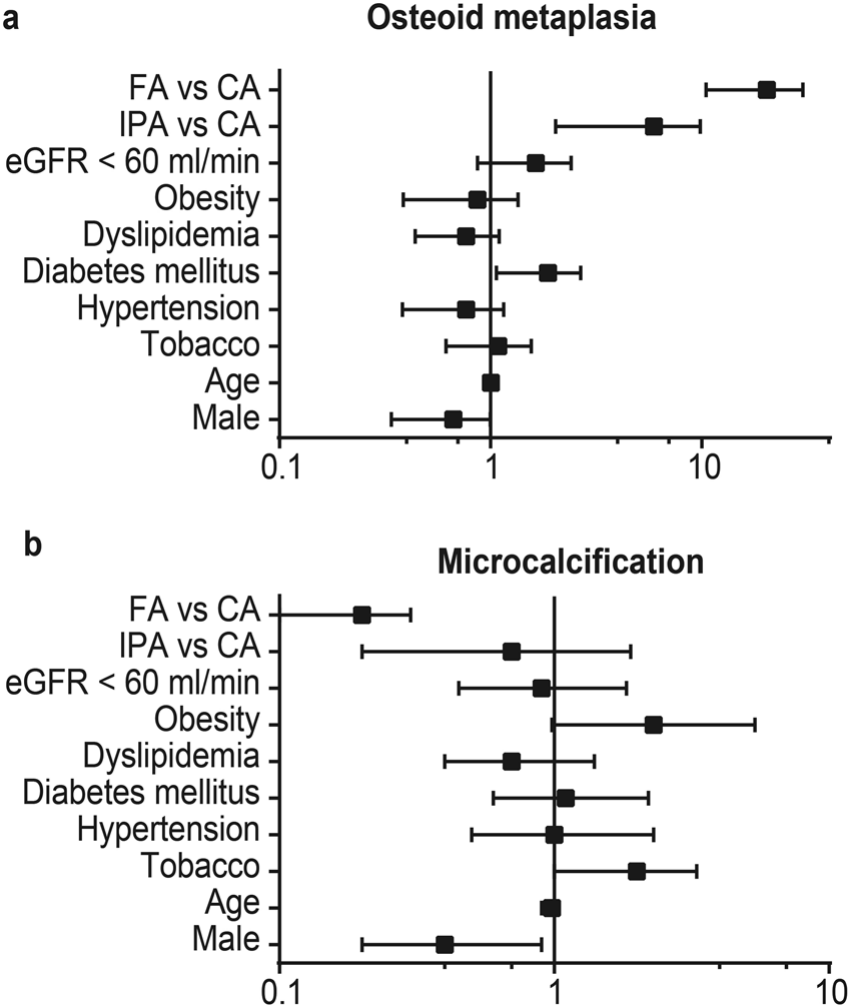
Forrest plot of relationships between cardiovascular risk factors, arterial territories and osteoid metaplasia (**a**) or microcalcification (**b**). Arterial territories influence the type of plaque calcification, whereas cardiovascular risk factors are not associated with the type of calcification. (CA: carotid arteries, FA: femoral arteries, IPA: infrapopliteal arteries, eGFR: estimated glomerular filtration rate)

Multivariate analysis further confirmed that arterial bed influences the presence of osteoid metaplasia, as FA developed drastically more osteoid metaplasia than CA or IPA (p < 0.0001) (Fig. 2). Sheet and nodular calcifications also varied among arterial beds; FA presented more sheet (p < 0.0001) and more nodular calcification (p < 0.0001) than CA.

### K-means clustering of arterial territories

**Figure 3:**
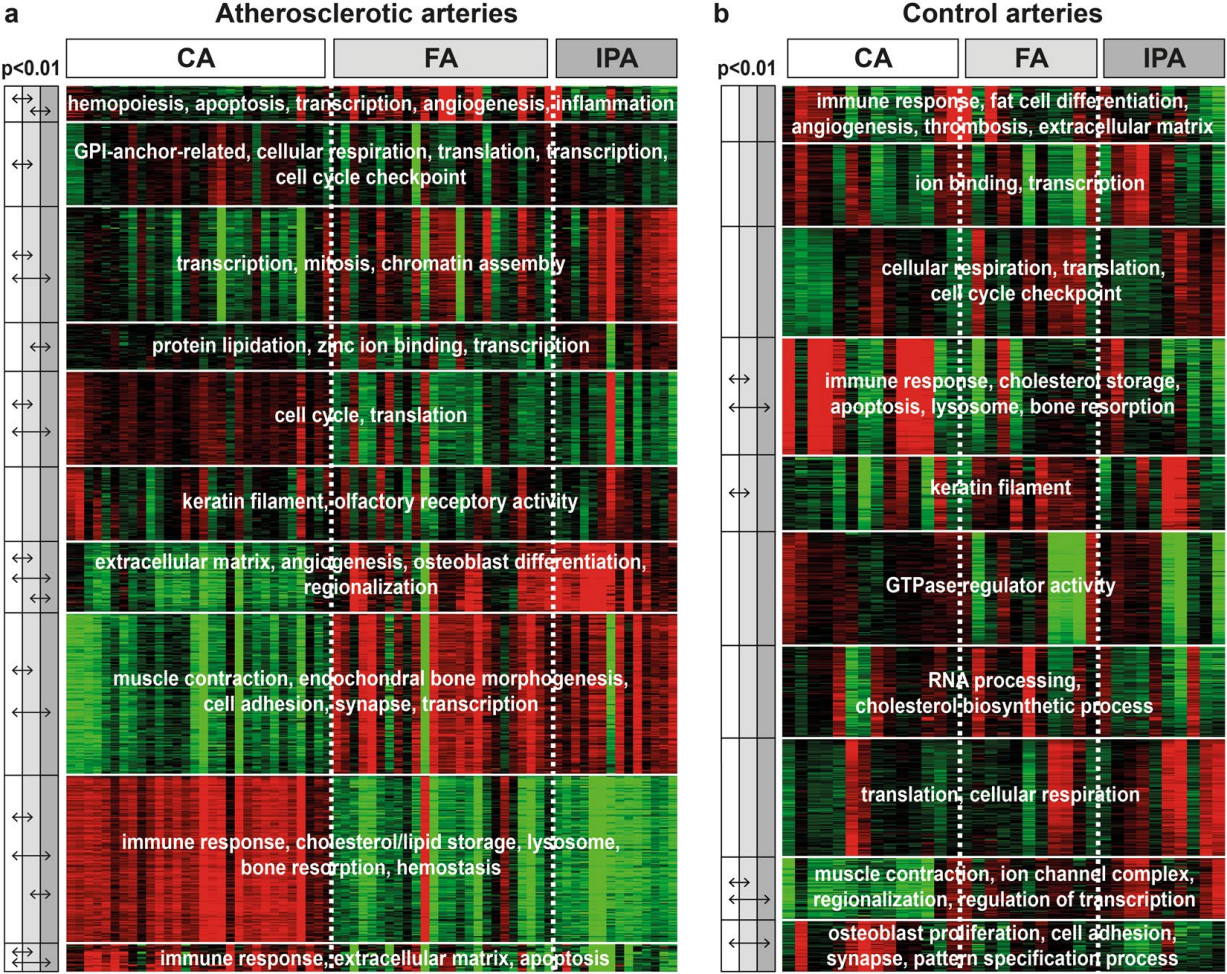
K-means clustering and GO enrichment analysis of (**a**) atherosclerotic and (**b**) healthy arteries. Gene expression is presented as a colored matrix in which each row represents a gene and each column represents a sample. Green, black and red correspond to lower values, median values and higher values, respectively. Representative enriched Gene Ontology terms (FDR < 5%) are indicated within each cluster. Within each cluster, the average of all probes was calculated for each sample. These average values were then compared between the arterial territories with Student’s t-test. Inter-territorial comparisons with p < 0.01 are indicated by horizontal arrows on the left side of the clusters. White = CA = carotid artery; light gray = FA = femoral artery; dark gray = IPA = infrapopliteal artery.

CVRF affect plaque development differently between arterial beds, but they have minor effects on differential calcification in atherosclerosis lesions. We explored the hypothesis that calcification heterogeneity might be directly derived from intrinsic vascular bed heterogeneity by performing transcriptome analysis on atherosclerotic and healthy arteries from the CA, FA, and IPA territories. We first obtained an overview of the functional annotations associated with each arterial territory by using k-means clustering combined with Gene Ontology enrichment analysis. Atherosclerotic lesions display clear arterial-territory-associated gene expression profiles (Fig. 3a). Of the ten clusters, all but one cluster (cluster 6) display differential expression between at least two arterial territories. In addition, all clusters were significantly enriched in functional annotations. Clusters 7 to 10 show the most extreme expression differences, clearly distinguishing CA vs. FA and IPA. Genes highly expressed in CA (clusters 9 and 10) were significantly enriched in functional annotations related to immune response, lipid storage, lysosomal functioning, bone resorption, hemostasis, extracellular matrix and apoptosis. Genes highly expressed in FA and IPA (clusters 7 and 8) were significantly enriched in functional annotations related to extracellular matrix, angiogenesis, osteoblast differentiation, regionalization, muscle contraction, endochondral bone morphogenesis, cell adhesion, synapse and transcription. Although the expression profiles of atherosclerotic FA and IPA were similar, some functions appear to be differently affected in both arterial territories. Genes involved in hematopoiesis, apoptosis, transcription, angiogenesis and inflammatory response (cluster 1) were more highly expressed in FA than in IPA. The opposite was observed for genes involved in protein lipidation, zinc ion binding and transcription (cluster 4). In healthy arteries without atherosclerotic lesions, we also identified associations between arterial territories and functional annotations, albeit to a lesser extent (Fig. 3b). As in the pathological arteries, genes associated with immune response, cholesterol storage, apoptosis, lysosome and bone resorption (cluster 4) were more highly expressed in CA than in FA and IPA, whereas genes involved in muscle contraction and regionalization had lower expression in CA than in FA and IPA (cluster 9). Differences between the pathological and healthy tissues were also observed. In healthy arteries, functional annotations associated with the genes from cluster 10 (including osteoblast proliferation and pattern specification process) were affected by up-regulation in CA compared to FA and IPA. This result was not found in pathological arteries.

### Differential gene expression analysis of arterial territories

**Figure 4:**
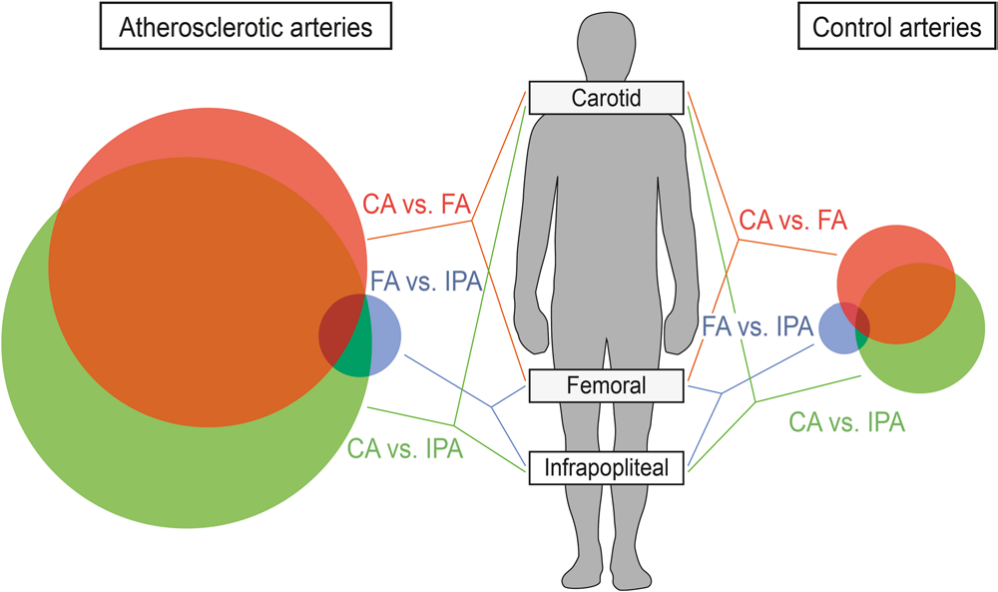
Proportional Venn diagram of differentially expressed genes between 3 arterial territories in atherosclerotic and healthy arteries according to two-class SAM analysis^50^.

Two-class SAM analysis (FDR = 0) between the different arterial territories identified a large number of differentially expressed genes in atherosclerotic arteries. The number of differential genes seemed to be proportional to the anatomical distance between the arterial territories: 5,181 genes between CA and IPA, 3,836 genes between CA and FA and 257 genes between FA and IPA. As shown schematically in Fig. 4 (left side), genes that were differentially expressed between CA and FA largely overlapped those that were differentially expressed between CA and IPA. In healthy arteries, we also identified genes that were differentially expressed between the three arterial territories (Fig. 4, right side). Again, the number of differential genes seemed proportional to the anatomical distance: 215 genes between CA and IPA, 201 genes between CA and FA and 8 genes between FA and IPA. For atherosclerotic arteries, anatomical distance was reflected by a substantial overlap between genes that were differentially expressed between CA and IPA and between CA and FA. However, this overlap was relatively smaller in healthy arteries than in atherosclerotic arteries, pointing towards a more territorial-specific gene expression profile in healthy arteries. All genes are listed in Supplementary File 1.

**Figure 5:**
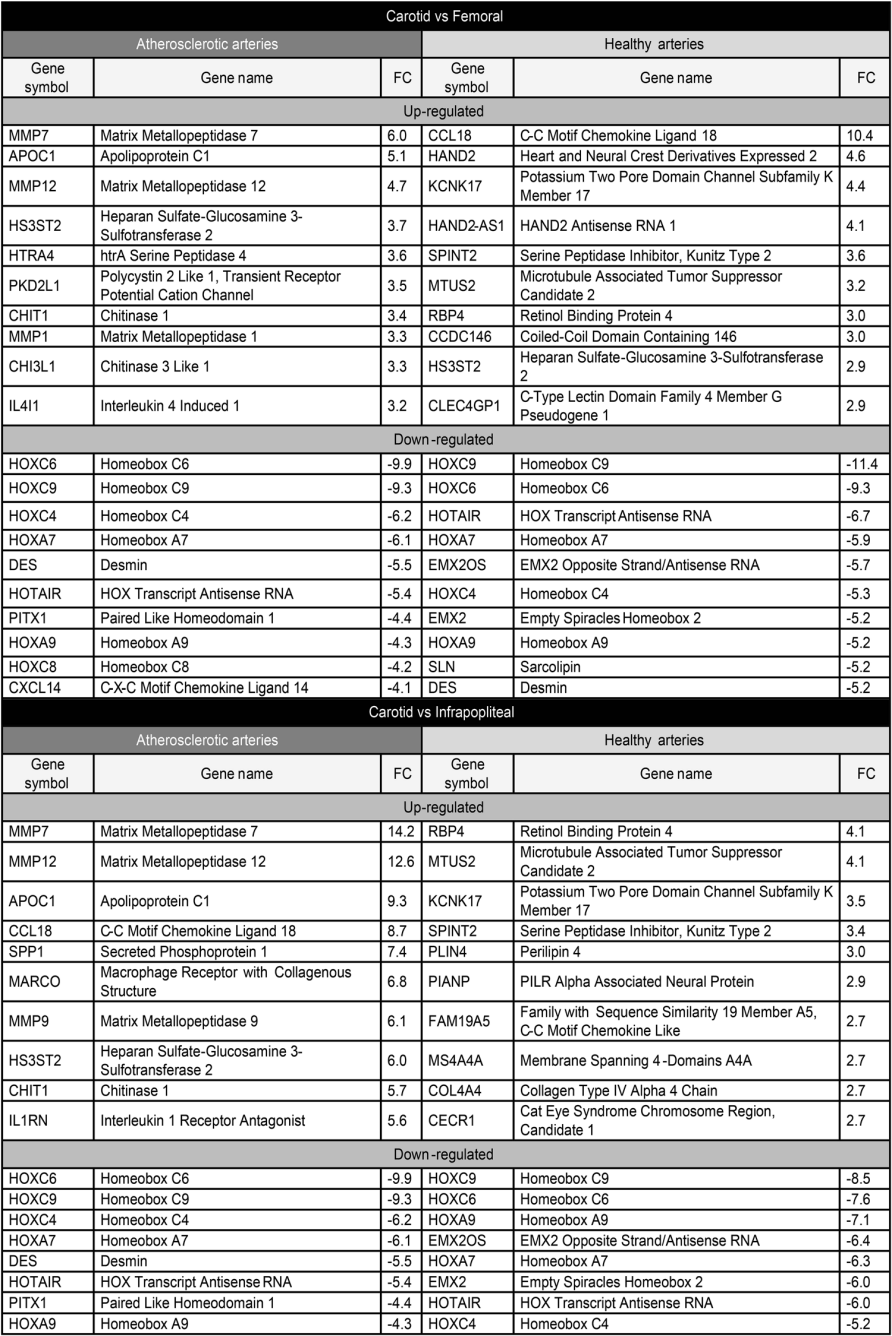
Top 10 most differentially expressed genes between arterial territories in atherosclerotic and healthy arteries after SAM analysis (FDR = 0%). (IPA: infrapopliteal arteries, FC: fold changes, FDR: false discovery rate).

Figure 5 shows the top 10 most differentially expressed genes between the three arterial territories in both atherosclerotic and healthy arteries. For atherosclerotic arteries, the results were very similar for the comparisons between CA and FA and between CA and IPA: compared to both FA and IPA, CA displayed higher expression levels of several matrix metallopeptidases (MMP7, MMP12, MMP1 (CA vs. FA only) and MMP9 (CA vs. IPA only)) and lower expression levels of several homeobox genes (HOXA7, HOXA9, HOXC4, HOXC6, HOXC8 and HOXC9). The FA vs. IPA comparison revealed higher expression levels of AP-1 transcription factor complex components (FOSB, FOS and ATF3) in FA and lower expression levels of two bone-related genes (STMN2 and CHI3L2) and two angiogenesis-related genes (ISM1 and SFRP1). Among the top 10 most differentially expressed genes in healthy tissue, four genes were expressed at a higher level in CA than in both FA and IPA: KCNK17, SPINT2, MUTS2 and RBP4. CCL18, which is involved in inflammatory and immune responses, was the most differential gene in CA vs. FA. Perilipin 4 (PLIN4), which is involved in the biogenesis of lipid droplets, was among the genes that were expressed at a higher level in CA vs. IPA. As in atherosclerotic arteries, several homeobox genes (HOXA7, HOXA9, HOXC4, HOXC6, HOXC9, and EMX2) were expressed at a lower level in CA vs. both FA and IPA in healthy arteries. Gene expression differences between healthy FA and IPA resembled those between atherosclerotic FA and IPA with the involvement of AP-1 transcription factor complex components (more highly expressed in FA) and vascular-development-associated gene HAND2 (more highly expressed in IPA).

**Figure 6:**
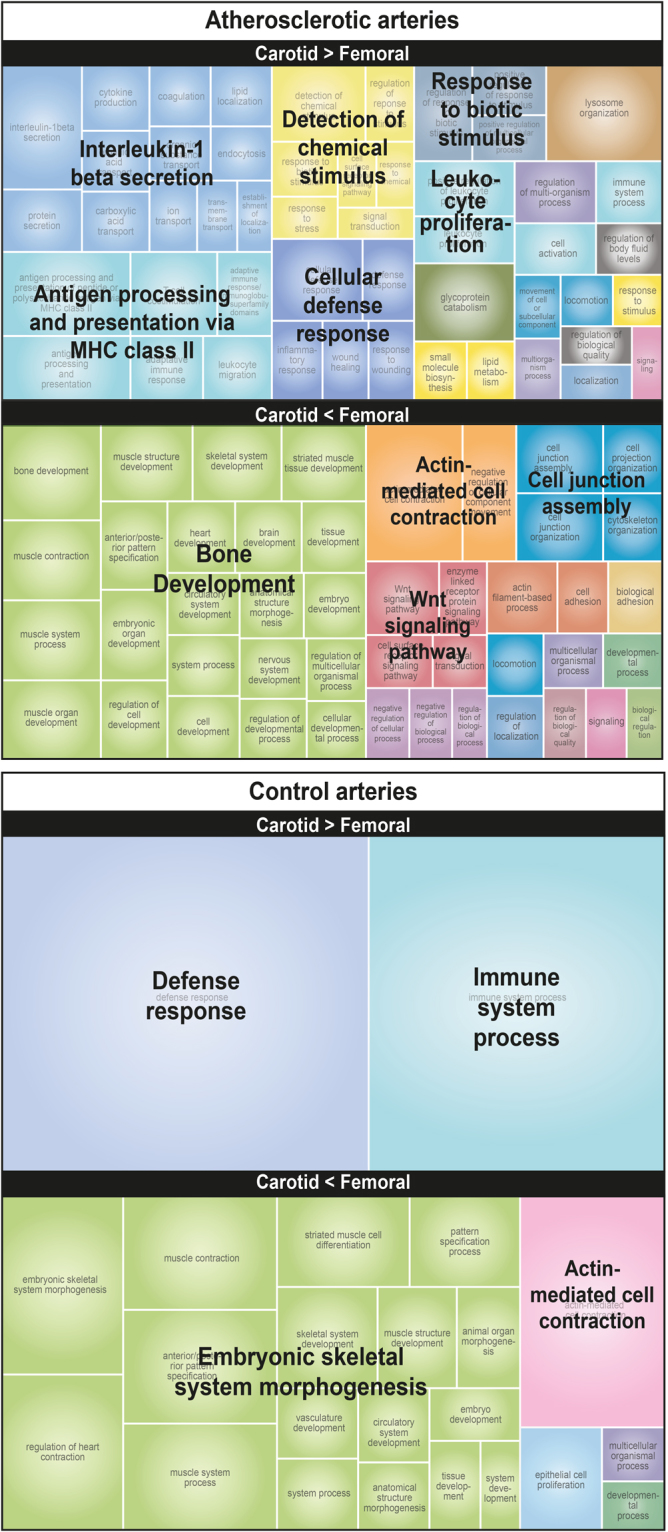
REVIGO TreeMap view of GO terms enriched among up- (upper TreeMaps) or down-regulated (lower TreeMaps) genes in CA vs. FA in atherosclerotic and healthy tissue. Input consisted of GO terms enriched at FDR = 0% for atherosclerotic arteries or FDR < 5% for healthy arteries with at least 5 changed genes per GO category. Each rectangle is a single cluster representative. The representatives are joined into ‘superclusters’ of loosely related terms that are visualized with distinct colors (indicated by centralized black text). The size of the rectangles reflects the enrichment of the GO term.

Figure 6 displays an overview of functional annotations enriched among differentially expressed genes between CA and FA. Because of the limited number of genes differentially expressed between FA and IPA, we did not include IPA in this analysis. In atherosclerotic tissue, genes that were more highly expressed in CA than in FA are characterized by their association with inflammatory processes, including innate and adaptive immunity, such as interleukin-1 production and antigen presentation. Genes that were more highly expressed in FA than in CA show a clear enrichment of bone-development-associated functional categories. In healthy tissue, the smaller number of genes differentially expressed between CA and FA somewhat limited the analysis. However, we were able to reveal an association between a higher expression in CA vs. FA and functional categories associated with the immune system. Genes that were more highly expressed in FA than in CA healthy tissue were mostly involved in muscle and skeletal system development. The full list of enriched GO terms is provided in Supplementary File 2.

### Calcification transcriptional signature of arterial territories

**Figure 7:**
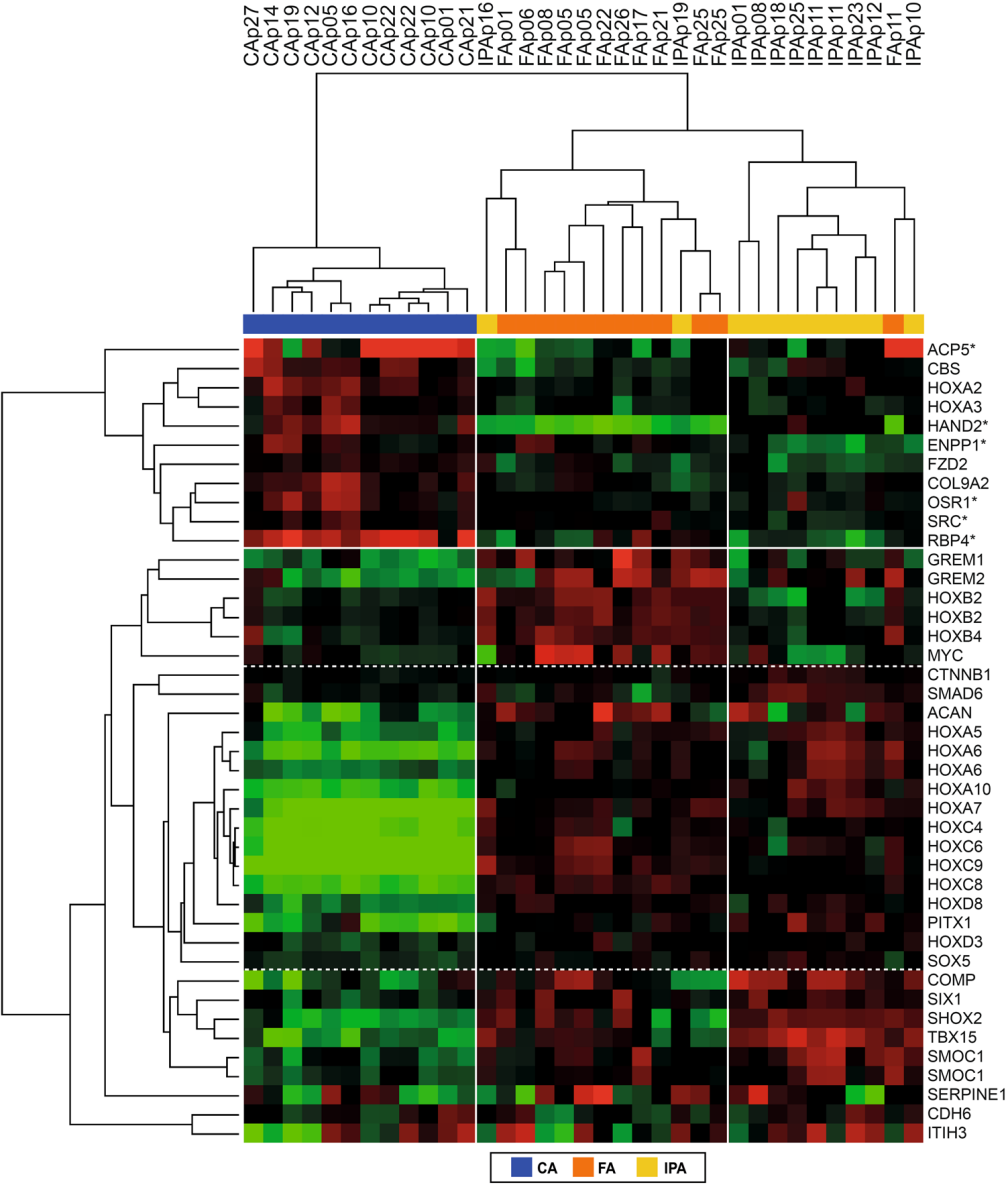
Two-way hierarchical clustering of control arteries and calcification-related genes. Genes were selected based on significant differential expression between the three arterial beds in control arteries (multiclass SAM analysis, FDR = 0%) and an association with bone-related functions through literature analysis or the presence of the terms ‘bone’, ‘cartilage’, ‘mineralization’ or ‘skeletal development’ in their GO categories. Gene expression is presented as a coloured matrix where each row represents a gene and each column a sample. Green, black and red correspond to lower values, median values and higher values, respectively. Colour coding of the samples is as follows: blue = carotid arteries, orange = femoral arteries, yellow = infra-popliteal arteries. The main branches of the gene and sample clusters are separated by white lines.

To further focus on the association between arterial territories and differential calcification, we selected genes involved in calcification-related functions from those differentially expressed among the three arterial territories in control samples based on multiclass SAM analysis. A total of 39 genes were selected based on the presence of the terms ‘bone’, ‘cartilage’, ‘mineralization’ or ‘skeletal development’ in their GO categories or on literature analysis. Figure 7 shows that two-way hierarchical clustering based on these 39 calcification-related genes separated the control arteries into three groups: One group – very distinct from the other two groups - consisting only of carotid arteries, one group consisting mainly of femoral arteries, and one group consisting mainly of infra-popliteal arteries. Two main gene expression profiles – separated by a solid white horizontal line in Fig. 7 - are responsible for this separation: A group of 11 genes with a significant higher expression level in CA than in FA and IPA, and a group of 28 genes with an inverse profile. Among these 39 genes, six (denoted with an asterisk in Fig. 7) have been associated with negative effects on bone formation and they all display a higher gene expression level in CA than in FA and IPA. HAND2 shows the most consistent lowest expression level in all FA samples. Dotted horizontal lines in Fig. 7 delineate gene clusters with differential expression between each arterial bed.

## Discussion

This study further demonstrates calcification plaque heterogeneity among peripheral arteries. Osteoid metaplasia is very frequent in FA, whereas microcalcifications are more prominent in CA. Our results show that plaque calcification types are not linked to CVRF, whereas CVRF specifically associate with peripheral territory in atherosclerosis. This finding is supported by previous studies showing that tobacco induces more lesions in arteries of the lower limbs than in cerebral or coronary arteries and that hypertension favors intracranial atheroma^9–11^. Older age, male sex, diabetes, heart failure, and critical limb ischemia are associated with distal disease, whereas female sex, smoking, hypertension, dyslipidemia, coronary heart disease, cerebrovascular disease, chronic obstructive pulmonary disease, and critical limb ischemia are associated with proximal disease. In patients with PAD, proximal and distal disease locations are associated with distinct risk factors and comorbidity profiles. Distal disease is associated with worse survival even after adjustment for risk factors, comorbidities, and resting ankle brachial index^9^. Accordingly, we report in our study that tobacco use was more frequent among patients undergoing femoral endarterectomy than among those who underwent carotid endarterectomy and that dyslipidemia was more frequent among the latter group.

Plaque calcification and morphology have also been reported to be linked to chronic kidney disease, insulin resistance and dyslipidemia in patients with PAD^36^. However, even though CVRF have an impact on the location of disease progression, they do not appear to have a significant impact on the type of calcification according to our findings; age and hypertension are the only CVRF associated with vascular calcification. Age potentially reflects the progression of disease, and hypertension may reflect the extent of calcification rather than act as a causal factor.

Altogether, these results further reveal a high degree of heterogeneity among arterial beds, as they are differentially associated with CVRF in atherosclerosis development, developing distinct plaques and calcification types^3,37,38^. Arterial biological heterogeneity, which may be directly responsible for these discrepancies, has also been repeatedly observed clinically. Long term outcomes after open surgery show drastic differences depending on the area treated. The infra renal aorta and its branches (including FA) has greater atherosclerosis recurrence than the aortic arch or the celiac aorta^20^. Stenting results also suggest arterial heterogeneity depending on the treatment area. Clinical studies have shown that rates of intra-stent restenosis vary considerably from one arterial location to another: the frequency of restenosis at one year is between 6 and 9% at the carotid level, 20% at the common femoral level and between 30 and 40% at the superficial femoral level^21,39–43^. For arteries of smaller caliber, such as leg arteries, the intra-stent restenosis rates (30–50%) exceed those observed in the coronary artery (10–35%)^22,44^. Moreover, restenosis progression differs between arterial beds. The risk of restenosis in coronary arteries has been shown to reach a plateau at six months^45^. For FA, the plateau occurs between 12 and 18 months^21^.

Arterial heterogeneity has thus been known for years, but little data are available at molecular level as evidence to support this notion. We are the first to identify transcriptomic differences according to peripheral arterial beds for both atherosclerotic and healthy human arteries. Sulkava *et al*. analyzed the differential expression of major pathways by comparing different pathological territories (CA, FA and abdominal aorta) to healthy internal thoracic arteries^25^. Arterial territory-associated gene expression changes were studied in only atherosclerotic arteries. In agreement with their results, we identified several HOX genes that were expressed at a significant lower level in atherosclerotic CA vs. FA. Some overlap (including HOXA2) was also identified between both gene lists with significantly higher expression levels in atherosclerotic CA vs. FA. However, in contrast to their findings, we identified higher expression of several MMPs in CA vs. FA. The comparison between both studies was limited by the fact that Sulkava *et al*. only included genes in this analysis that were differentially expressed in pathological vs. healthy arteries. Furthermore, they did not directly compare the implication of canonical pathways in atherosclerosis in different arterial beds; instead, they only listed pathways that they found to be involved in pathological vs. healthy arteries. Therefore, their study provided ample results on genes involved in atherosclerosis in general, but did not provide results on inter-territorial differences. Our study focused on the differences between carotid, femoral and infrapopliteal arterial beds in both atherosclerotic and healthy tissue. Gene Ontology enrichment analysis of k-means clusters clearly reflected the known histological characteristics of carotid vs. femoral atherosclerotic plaques^3^. The association of femoral plaques with the presence of bone-related features was illustrated by an enrichment of genes involved in osteoblast differentiation and bone morphogenesis in these plaques. Examples include the high expression levels of transforming growth factor beta 3 (TGFB3) and bone morphogenetic protein 2 (BMP), which have a synergistic effect of osteogenic differentiation^46^. BMP2 kinase (BMP2K), which may impair osteoblast differentiation, had reduced expression in femoral plaques^47^. Two structurally related members from the thrombospondin family (COMP and THBS3) were also among the femoral-plaque-specific genes. Both of these extracellular matrix genes are involved in bone development, and COMP has been shown to be stimulated by TGFB3^48^. The enrichment of immune response and lipid-related functional categories in carotid atherosclerotic arteries corresponded with the known enrichment of lipids and inflammatory cells in these plaques. In our analysis focused on the identification of genes that were significantly differentially expressed between diseased arterial beds, we consistently found key players in inflammation, matrix remodeling (MMPs), and numerous HOX family genes. HOX genes encode transcription factors that play a key role in morphogenesis. Genes regulated by HOX transcription factors are involved in development and skeletal differentiation.

These results most likely reflect the major differences in composition observed histologically between lipid-rich and inflamed lesions in CA compared to fibrotic and heavily calcified plaques in FA^25,49^. Our study identified gene expression changes between non-atherosclerotic arterial tissue from different arterial territories. These results now allow us to distinguish between genes involved in atherosclerosis at the clinical stage of the disease (e.g., metalloproteinases) and genes potentially involved in differential predisposition to the disease (HOX genes and the AP-1 transcription factor complex). The differential representation of functional categories among genes expressed in different arterial beds in healthy arteries displayed striking similarities to those in atherosclerotic arteries. In both cases, genes involved in the immune system are more strongly expressed in CA than in FA, whereas genes involved in skeletal (bone) development and muscle function are more strongly expressed in FA than in CA. These results are consistent with the type of lesions typically found in these locations. This important finding suggests that arterial heterogeneity precludes the evolution of plaque fate and may be critical in predisposing arteries to the development of fibrosis and calcification.

## Electronic supplementary material

Supplementary information

Supplementary file 1

Supplementary file 2
