# Supplementary material for: Identification of genomic differences among peripheral arterial beds in atherosclerotic and healthy arteries
Source: Sci Rep. 2018 Mar 2;8:3940. doi: 10.1038/s41598-018-22292-y (PMC5834518; doi:10.1038/s41598-018-22292-y)
Supplement: Supplementary file 1 — Supplementary information [file 41598_2018_22292_MOESM1_ESM.docx]

**Identification of genomic differences among peripheral arterial beds in atherosclerotic and healthy arteries**

Marja Steenman^2*^, Olivier Espitia^1,3*^, Blandine Maurel^1,4^, Beatrice Guyomarch^2^, Marie-Françoise Heymann^8^, Marc-Antoine Pistorius^3^, Benjamin Ory^1^, Dominique Heymann^7,8^, Rémi Houlgatte^5,6^, Yann Gouëffic^1,4^ and Thibaut Quillard^1^

**Supplementary materials list:**

**Supplementary file 1**. List of all genes significantly differentially expressed between carotid, femoral and infrapopliteal control arteries.

**Supplementary file 2**. The full list of enriched GO terms for the differential gene expression between diseased and control carotid and femoral arteries, respectively
